# Decoherence of Hyperfine Coupled ^19^F and ^1^H Nuclei in Gadolinium(III) Model Complexes

**DOI:** 10.1021/acs.jpcb.5c03224

**Published:** 2025-06-21

**Authors:** Alexey Bogdanov, Veronica Frydman, Xun-Cheng Su, Daniella Goldfarb

**Affiliations:** † Department of Chemical and Biological Physics, 34976 The Weizmann Institute of Science, P.O. Box 26, Rehovot 7610001, Israel; ‡ Department of Chemical Research Support, The Weizmann Institute of Science, P.O. Box 26, Rehovot 7610001, Israel; § State Key Laboratory of Elemento-Organic Chemistry, 12538 Nankai University, Tianjin 300071, P. R. China

## Abstract

Time-domain electron–nuclear double resonance (ENDOR) was employed to determine the nuclear decoherence rate (1/*T*_2n_) of fluorine nuclei in four model Gd­(III) chelates of varying structures, over the temperature range 3.7–10 K. These complexes are derivatives of Gd­(III) spin labels used in ^19^F-ENDOR distance measurements, which emerged as an efficient method for measuring short-range distances in biomolecules. It was found that the ^19^F decoherence originates primarily from electron spin flips, following the relationship *T*_2n_ ≈ 2*T*_1e_, where *T*_1e_ is the Gd­(III) spin–lattice relaxation time. The obtained ^19^F decoherence rates (1–8 kHz) indicate that the intrinsic ^19^F-ENDOR line widths are small relative to other broadening contributions, and therefore do not significantly limit distance resolution of ^19^F ENDOR. Proton decoherence measurements were carried out for comparison, revealing two ^1^H populations with different relaxation rates; the faster component was sensitive to the matrix protonation and attributed to dipolar flip-flops among weakly coupled protons, a mechanism absent for ^19^F. These results elucidate key factors affecting ENDOR resolution and provide new insights into the nuclear spin relaxation mechanism in paramagnetic systems.

## Graphical abstract

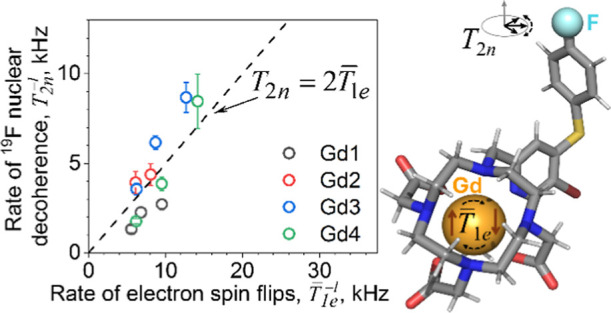

## Introduction

^19^F electron–nuclear double resonance (^19^F-ENDOR) spectroscopy has recently attracted considerable attention as a tool for measuring short nanometer-range distances (10–20 Å) in nucleic acids,^1–7^ proteins^2,8–11^ and molecular materials.^12–14^ It complements pulse dipolar EPR techniques that commonly provide distances in the range of 20–80 Å.^15,16^ In ^19^F-ENDOR, a ^19^F label and an electron-spin label are introduced at chosen positions within the molecule of interest, and the electron–nuclear distance is derived from the measured dipolar hyperfine splitting in the ENDOR spectrum.^1^ A wide range of spin labels can be used,^11^ including organic radicals, most commonly, nitroxide^1,6,7^ and trityl,^4^ as well as metal ions, such as Gd­(III)^8,10^ or Cu­(II).^5^ Notably, Gd­(III) spin labels have proven particularly advantageous due to the enhanced sensitivity and resolution they provide in ^19^F-ENDOR measurements, owing to the high electron spin of Gd­(III).^8–11,14,17^ In addition, their redox stability allows for in-cell ^19^F-ENDOR measurements.^9^

The longest electron–nuclear distance that can be accessed by ^19^F-ENDOR spectroscopy is primarily limited by the resolution and sensitivity of the measurements. In particular, the resolution of pulsed ENDOR spectra is affected by line broadening, which arises from several mechanisms. Theoretical^18^ and experimental^19^ studies have identified the major contributions to the line broadening as (i) distribution of electron–nuclear distances; (ii) radiofrequency (RF) pulse excitation bandwidth; (iii) chemical shift anisotropy of the probed ^19^F nucleus; (iv) dipolar interaction of the ^19^F with adjacent nuclei, and (v) the coherence time (transverse nuclear spin relaxation), *T*_2n_ of the ^19^F nucleus. The latter determines the highest possible resolution limit of the technique.

Due to insufficient experimental results, the intrinsic line width, determined by 1/*T*_2n_, remains one of the least understood factors influencing ^19^F-ENDOR resolution. Most information available on nuclear decoherence in paramagnetic systems comes from paramagnetic NMR, which is usually carried out at temperatures significantly higher than those typically used for ENDOR.^20^ This is a notable difference because at these low temperatures (4–50 K), the electron spin–lattice relaxation rate, 1/*T*_1e_, which is a major factor affecting 1/*T*_2n_, slows significantly. So far, there has been one report of the ^19^F’s *T*_2n_ in the presence of a nitroxide spin label at one temperature,^19^ and possible factors that govern the nuclear spin decoherence under ENDOR conditions, i.e., low-concentration of glassy solutions of paramagnetic species at low temperatures, are still not fully understood.

To fill this gap, in this work, we present measurements of *T*_2n_ of ^19^F nuclei coupled to Gd­(III) in four model compounds **Gd1–Gd4** (see Figure 1A). These differ by the Gd­(III) chelates, (DO3A or PyMTA, both commonly used in Gd­(III) spin labels) and the fluorine substituents. We analyzed the data in terms of possible mechanisms accounting for the dependence of 1/*T*_2n_ on 1/*T*_1e_ and on the Gd­(III) distance. Finally, we compared the results to the respective behavior of hyperfine-coupled ^1^H nuclei in the same systems, where nuclear spin diffusion, not present for ^19^F, may play a significant role in decoherence.

**Figure 1:**
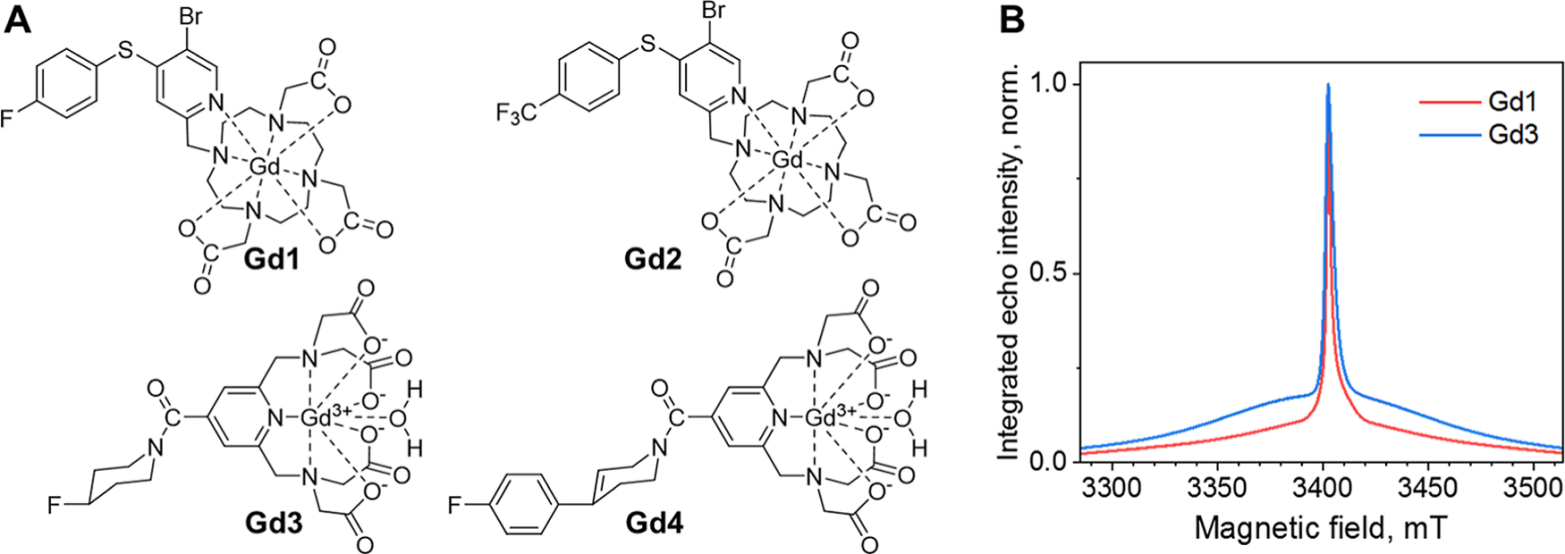
(A) Chemical structures of the fluorinated Gd-DO3A complexes (**Gd1**, **Gd2**) and Gd-PyMTA complexes (**Gd3**, **Gd4**) studied in the present work. (B) Normalized echo-detected W-band electron paramagnetic resonance (ED-EPR) spectra of **Gd1** and **Gd3**, recorded at 10 K.

## Theoretical Background

It is well established that a major contribution to transverse and longitudinal nuclear relaxation in solids arises from interaction with paramagnetic centers.^21–25^

The transverse nuclear relaxation rate in the presence of a paramagnetic center with a fast electron spin–lattice relaxation rate compared to the hyperfine coupling, 1/*T*_1e_ ≫ *a*_⊥_ in the absence of motion is usually treated in the framework of the Solomon–Bloembergen–Morgan theory, which gives^25,26^

1 $$\frac{1}{T_{2n}}=\frac{{\left(2\pi a_{⊥}\right)}^{2}}{15}S\left(S+1\right)\left[4T_{1e}+\frac{3T_{1e}}{1+\omega_{I}^{2}T_{1e}^{2}}\right]$$

where *S* is the electron spin, *a*_⊥_ is the perpendicular principal component of the dipolar hyperfine splitting tensor in cyclic frequency units, and ω_I_ is the nuclear Larmor frequency. In eq 1 terms on the order of *T*_2e_/(1 + ω_e_^2^ *T*_2e_^2^) and $T_{2e}/\left(1+{\left(\omega_{e}\pm \omega_{I}\right)}^{2}T_{2e}^{2}\right)$ , where ω_e_ is the electron Larmor frequency and *T*_2e_ is the electron transverse relaxation time, were omitted because of their negligible contributions. Under these conditions, the NMR hyperfine doublet collapses into a single line, the line width of which is given by eq 1. At the other extreme, where 1/*T*_1e_ ≪ *a*_⊥_ ^27^

2 $$\frac{1}{T_{2n}}=\frac{1}{2T_{1e}}$$

This was derived for *S* = 1/2 and demonstrated on a ^31^P-doped ^28^Si-enriched silicon single crystal, where *T*_2n_ was found to approximately follow *T*_1e_ over the range 9–12 K. In this case, the NMR hyperfine doublet remains resolved, and the line width of each component is given by eq 2.

Whereas the validity limits of eqs 1 and 2 correspond to fast and slow regimes of 1/*T*_1e_ with respect to the hyperfine splitting, respectively, for the intermediate regime, where 1/*T*_1e_ is on the order of *a*_⊥_, analytical expressions have not been reported.

## Experimental and Computational Details

### Synthesis of Compounds

#### **Gd1** and **Gd2**

A solution of BrPSPy-DO3A-Gd­(III), synthesized as described earlier,^28^ in 20 mM deuterated phosphate buffer (pD = 6.5–7.0) (150–300 μM), was mixed with a 5-fold excess of 4-fluoro thiophenol or 4-trifluoromethyl thiophenol (abcr GmbH, Germany) in the same buffer. The reaction mixture was stirred for 6 h at 5 °C. The adducts were used without further purification, as excess fluorothiophenol or 4-trifluoromethyl thiophenol does not contribute to the ENDOR signal. **Gd3** was synthesized as previously described.^10^ **Gd4** was produced using the synthetic strategy outlined in Scheme S1. The details of the synthesis procedures and analyses are given in the Supporting Information.

The purity and identity of all products were confirmed based on high-resolution mass spectra, recorded on a Waters SYNAPT XS time-of-flight ion mobility high resolution mass spectrometer equipped with liquid chromatography infusion unit and electrospray ionization. The high-resolution mass spectra and isotope fits of all compounds are presented in Figure S1.

### Sample Preparation for ENDOR Measurements

Pulse EPR and ENDOR spectra of **Gd1** and **Gd2** were recorded on samples containing 75:25 D_2_O:glycerol-d_8_ (v/v). To determine the effect of solvent protonation on the nuclear relaxation, **Gd1** solutions in 10:65:25 H_2_O:D_2_O:glycerol-d_8_ (v/v) were prepared as well. Both solutions had the same **Gd1** concentration. The samples of **Gd3** and **Gd4** were prepared by dissolving the solid complexes in 1:1 D_2_O:glycerol-d_8_.

All samples were placed into 0.60 mm I. D. fused silica tubes sealed with crytoseal.

### Spectroscopic Measurements

Pulsed EPR and ENDOR measurements were performed using a home-built W-band pulsed EPR spectrometer equipped with a cylindrical TE_011_ cavity and a Helmholtz radiofrequency coil.^29^ The spectrometer has a 0–5 T cryogen-free magnet with an integrated variable temperature unit and 300 mT sweep coil (J3678, Cryogenic Ltd.). It is equipped with a 2 W pulsed microwave power amplifier (QPP95023330-ZW1, Quinstar) and a pulsed 2 kW RF amplifier (BT02000-GammaS, TOMCO). The RF pulses were produced using an arbitrary-wave generator (DAx22000, Wavepond).

Echo-detected electron paramagnetic resonance (ED-EPR) spectra were recorded using the Hahn echo (π/2 – τ – π – τ – echo) sequence. Mims ENDOR spectra were recorded using the sequence π/2 – τ – π/2 – *T*(π_RF_) – π/2 – τ – echo – [τ_2_ – π – τ_2_ – echo – τ_2_ – –π – τ_2_ – echo]_*n*_ with a four-step phase cycle and a Carr–Purcell (CP) detection train at the end to enhance the signal-to-noise ratio.^30^ We used three to five CP echoes with τ_2_ = 600 ns for detection. Each echo was integrated over a 20 ns window, optimized for the best signal-to-noise ratio. Random sampling of RF was employed,^31^ with 5–10 shots acquired per frequency point in each scan. Microwave power was adjusted to give π pulses of 28–32 ns, using the Rabi nutation sequence, *t*_nut_ – *t*_wait_ – π/2 – τ – π – τ – echo (*t*_nut_ was varied; *t*_wait_ was chosen such as to let for the decay of the transverse magnetization). RF power was adjusted to yield the desired π_RF_ pulse length, using a Rabi nutation sequence π/2 – τ – π/2 – *T*(*t*_RF_) – π/2 – τ – echo, with a constant mixing time *T* of 100 μs and varying RF pulse length, *t*_RF_. The RF pulse length was set to 35–60 μs. The mixing time *T* in the Mims ENDOR experiment was set to be 2–5 μs longer than the RF pulse length.

Time-domain Mims ENDOR nuclear spin echo (NSE) measurements were recorded using the sequence π/2 – τ – π/2 – *T*(π_RF_/2 – τ_1,RF_ – π_RF_ – τ_2,RF_ – π_RF_/2) – π/2 – τ – echo – [τ_2_ – π – τ_2_ – echo – τ_2_ – –π – τ_2_ – echo]_*n*_. The phase cycle for the microwave pulses was the same as in the frequency-domain Mims ENDOR measurement, and an additional four-step phase cycle on the RF pulses was used (see Section S3 in the Supporting Information).

Electron spin–lattice relaxation times, *T*_1e_, were measured using the saturation and inversion recovery pulse sequences, (*p*_HTA_) – *t*_wait_ – π/2 – τ – π – τ – echo for saturation recovery, and π – *t*_wait_ – π/2 – τ – π – τ – echo for inversion recovery. Here (*p*_HTA_) represents a high-turning angle saturation pulse (length 100–300 μs). The experiment was performed by measuring integrated echo intensity as a function of *t*_wait_. The recovery traces were fitted with a stretched exponential function

3 $$I_{echo}=I_{0}\left(1-A\cdot \exp\left(-{\left[t_{wait}/T_{1e}^{s,i}\right]}^{\beta^{s,i}}\right)\right)$$

where *T*_1e_^s,i^ is the spin–lattice relaxation time, β^s,i^ is the stretch parameter, and superscript “s” or “i” corresponds to saturation/inversion recovery. *A* ≈ 1 for saturation recovery and *A* > 1 for inversion recovery. β^s,i^ < 1 values indicate that there is a distribution of relaxation times.^32,33^ Examples of saturation and inversion recovery traces, their fitting, and the best fit *T*_1e_^s,i^ and β^s,i^ parameters are shown in Figure S2.

For quantitative comparison of relaxation times with varying stretch parameters, the averaged values of longitudinal relaxation times were taken as the first moment of the relaxation function

4 $${\mathrm{T̅}}_{1e}^{s,i}=\int_{0}^{\infty }\exp\left(-{\left[t/T_{1e}^{s,i}\right]}^{\beta^{s,i}}\right)\;dt=\frac{T_{1e}^{s,i}}{\beta^{s,i}}\cdot \Gamma \left(\frac{1}{\beta^{s,i}}\right)$$

where Γ(···) is the gamma function. This value represents the mean value of the distribution.^32^

### ENDOR Spectral Simulations

ENDOR spectral simulations were performed using programs and approaches published earlier.^10^ In brief, ENDOR resonance frequencies were calculated for the central transition of Gd­(III) (*m*_S_ = ±1/2) as

5 $$\nu =\nu_{I}\mp \frac{1}{2}a$$

where ν_I_ is the nuclear Larmor frequency, and *a* is the hyperfine splitting, which is orientation dependent. As we deal with long Gd–F distances in nonconjugated systems, *a* can be assumed to be purely dipolar according to

6 $$a\left(\theta \right)=\left(3\cos^{2}\theta -1\right)\frac{\mu_{0}g_{e}\mu_{B}g_{n}\mu_{N}}{4\pi hr_{GdF}^{3}}=\left(3\cos^{2}\theta -1\right)\left|a_{⊥}\right|$$

where θ is the angle between the magnetic field direction and the vector connecting the Gd­(III) ion and the ^19^F nucleus, μ_0_ is vacuum magnetic permeability, *g*_e_ and *g*_n_ are electron and nuclear *g*-values, μ_B_ and μ_N_ are Bohr magneton and nuclear magneton, respectively, *h* is the Planck constant, and **r**_GdF_ is the Gd–F distance.

When the ^19^F anisotropic chemical shift (CS) is resolved, also ν_I_ becomes orientation dependent. The asymmetric shape of the ^19^F-ENDOR spectrum of **Gd1** results from the resolved CS anisotropy, which must be considered in the simulations. It is given by the principal values of the anisotropic part of the CS tensor, δ_*xx*_, δ_*yy*_ and δ_*zz*_ and angles γ (polar) and ρ (azimuthal) that describe the direction of the Gd–F vector in the CS principal axis frame. If ξ (polar) and φ (azimuthal) are the angles that define the direction of the magnetic field in the CS frame, then

7 $$\nu_{I}\left(\xi ,\varphi \right)=\nu_{I,0}\left(1+\delta_{eff}\left(\xi ,\varphi \right)\right)$$

where ν_I,0_ is the reference ^19^F Larmor frequency and

8 $$\delta_{eff}\left(\xi ,\varphi \right)\approx \delta_{xx}\;\cos\varphi \;\sin\xi +\delta_{yy}\sin\varphi \;\sin\xi +\delta_{zz}\cos\xi$$

The angle θ is then given by

9 cos⁡θ=cos⁡ρsin⁡γcos⁡φsin⁡ξ+sin⁡ρsin⁡γsin⁡φsin⁡ξ+cos⁡γcos⁡ξ

In the simulations, integration over ξ and φ was performed to account for powder averaging.

The optimal values of the varied parameters were found using the nonlinear least-squares algorithm NL2SOL,^34^ and the parameter uncertainties were estimated from corresponding covariance matrices.

## Results and Discussion

### Echo Detected EPR and ^19^F and ^1^H ENDOR Spectra

All measurements were carried out at 95 GHz (W-band), which is an optimal frequency for high-spin Gd­(III). The echo-detected (ED) EPR spectra of **Gd1** and **Gd3** are shown in Figure 1B. **Gd2** and **Gd4** have the same spectra as **Gd1** and **Gd3**, because they have the same chelate. All measurements described in the present work were performed at the peak of the central Gd­(III) transition (CT), *m*_S_ = −1/2 ↔ *m*_S_ = +1/2, i.e. the field position corresponding to the maximum spectrum intensity.

The ^19^F-ENDOR spectra were measured using the Mims sequence (Figure 2A) which is suitable for measuring weak electron–nuclear couplings. The ^19^F-ENDOR spectra of **Gd1–Gd4** (Figure 3A), recorded at 10 K, consist of doublets separated by hyperfine splittings. The Gd–F distances, derived from simulations, are 10.0–10.5 Å for the **Gd1**–**Gd3** and 13.3 Å for **Gd4**. ^19^F-ENDOR spectra recorded at 3.7 K have essentially the same shapes (Figure S3). For Gd–F distances longer than that of **Gd4**, nuclear decoherence measurements could not be performed due to signal-to-noise restrictions.

**Figure 2:**
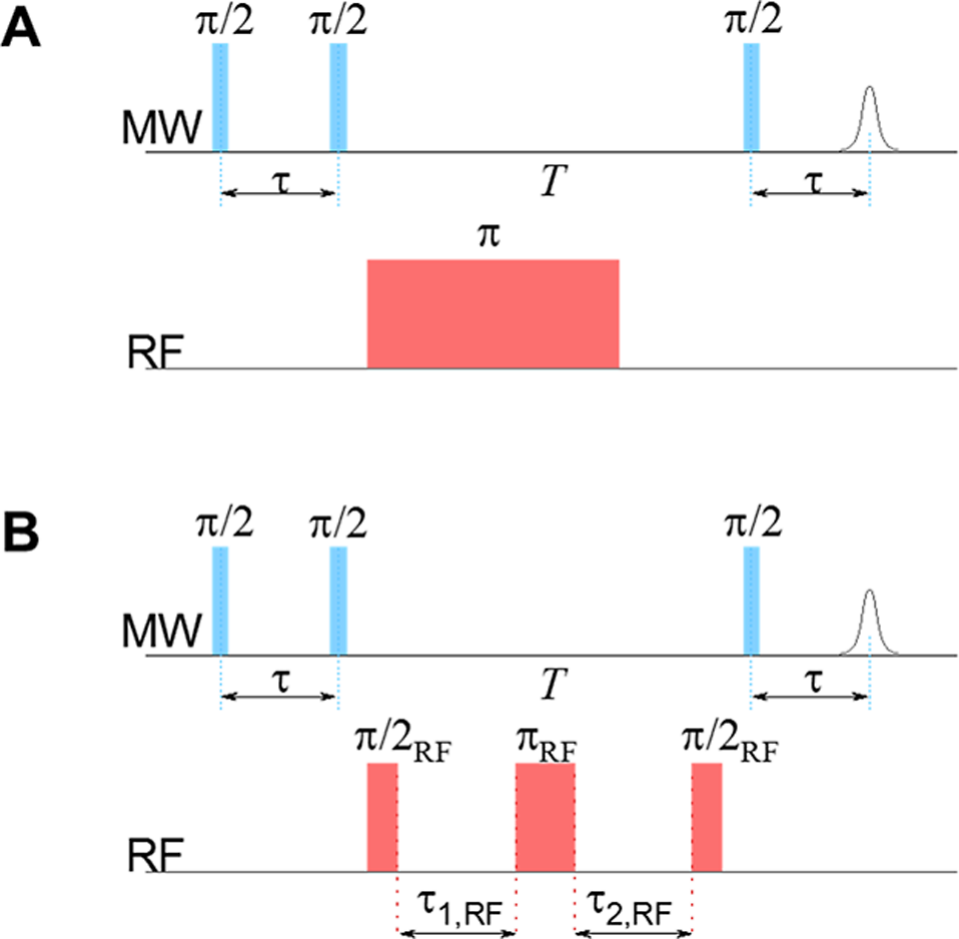
Pulse ENDOR sequences used in the present work: (A) Mims ENDOR, where the echo intensity is measured as a function of the RF pulse frequency. (B) Mims ENDOR for measuring the nuclear spin echo and it is decay, where the RF is constant and echo intensity is measured as a function of the τ_1,2,RF_ intervals.

**Figure 3:**
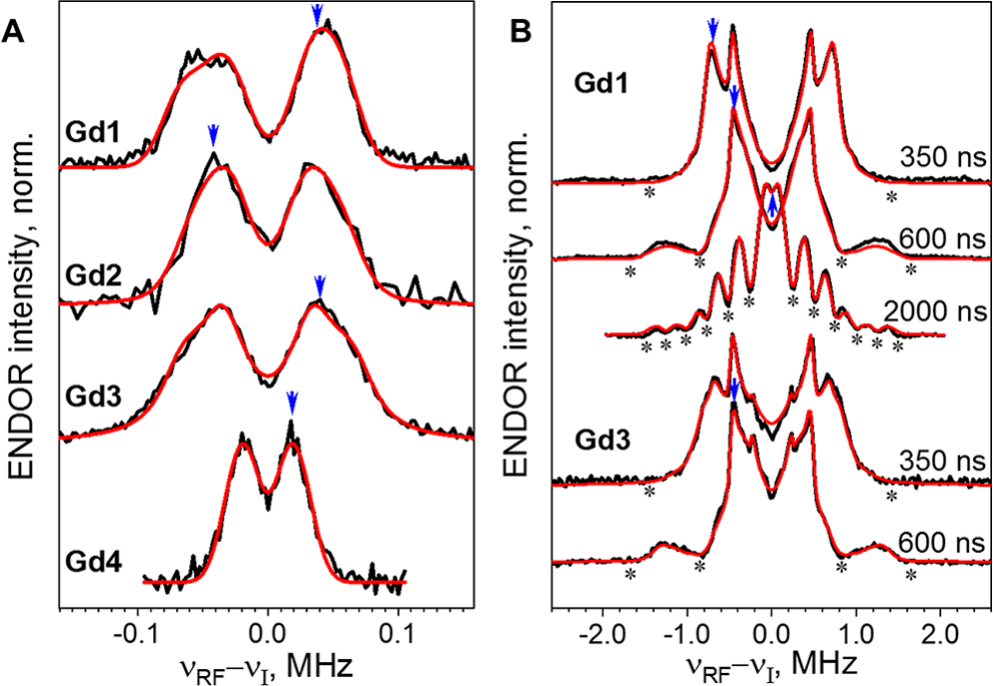
Experimental ^19^F-ENDOR spectra of **Gd1–Gd4** (A) and ^1^H-ENDOR spectra of **Gd1 and Gd3** (B) (black lines), recorded at 10 K, and their simulations (red lines). The parameters used in the simulations are listed in Tables S1 and S2. ^19^F spectra were recorded with τ = 2000 ns. ^1^H spectra were recorded with several τ-values, as noted in the figure. Blue arrows denote the frequency positions where NSE decays were measured, and asterisks mark the Mims blind spots.

The asymmetry of the spectrum for **Gd1** is due to the ^19^F chemical shift anisotropy, as confirmed by spectral simulation (see Figure 3A). Resolved ^19^F chemical shift anisotropy at 95 and 263 GHz was reported earlier for nitroxides.^12^ In the simulations of the **Gd1** spectrum, the components of the anisotropic CS tensor were taken from the literature for fluorobenzene^35^ (in ppm): (δ_*xx*_, δ_*yy*_, δ_*zz*_)=(98, −29, −69), while the values of angles γ, ρ were fitted, in addition to *r*_GdF_ and line widths, and were found to be 57° and 75°, respectively. This indicates that the Gd–F vector lies between the *y*- and *z*-axes of the CS tensor.

The ^19^F-ENDOR spectra of **Gd2** and **Gd3** do not show any asymmetry; namely, the ^19^F CS anisotropy is small and unresolved. This is consistent with the fluorine located at aliphatic positions, which exhibit smaller CS anisotropy than aromatic positions.^36^ The fluorinated groups in **Gd1** and **Gd4** have similar structures (*para*-substituted fluorobenzenes) and, therefore, the CS anisotropy should also be manifested in the ^19^F-ENDOR spectrum of **Gd4**. Nevertheless, the experimental spectrum is symmetric around ν_I_, and no effect of CS anisotropy is observed. A possible explanation is the relative orientation of the Gd–F vector and CS tensor in the molecular being close to the magic angle. To illustrate this, we present in Figure S4 the experimental spectrum of **Gd4** and two simulation results, one without considering CS anisotropy, and another with the same CS tensor as used for **Gd1**, and the angles (γ, ρ) = (39°, 0°). It shows that the two simulations produce almost identical fits and the same hyperfine splittings.

The tentative assignment of the well-resolved splittings in the ^1^H spectra of **Gd1** (proton types H1, H2) and **Gd3** (proton types H1–H5) (see Figure S5) was based on earlier work.^10^ To quantitatively account for the shape of ^1^H ENDOR spectra recorded at different τ values between 350–2000 ns, two types of weakly coupled protons (H3, H4) had to be taken into account for **Gd1**, and one type of weakly coupled protons (H6) for **Gd3**. These correspond to a combination of weakly coupled protons of the molecule and possibly residual protons of the medium.

### ^19^F Relaxation Measurements

A modification of the Mims ENDOR sequence is used to measure the nuclear spin echo (NSE) decay,^19,37,38^ as illustrated in Figure 2B. Replacing the inversion RF pulse (π_RF_) in the conventional, frequency domain, Mims ENDOR sequence (Figure 2A) by a block of three RF pulses, π_RF_/2 – π_RF_ – π_RF_/2 (Figure 2B), allows generating and monitoring the NSE within the excited nuclear sublevels. The generation of the NSE is illustrated in Figure 4A for ^19^F in **Gd1**; here, the first delay τ_1,RF_ is kept constant and τ_2,RF_ is swept to trace the NSE. A phase cycle on the RF part of the sequence was applied to ensure the elimination of unwanted nuclear spin evolution pathways (Figure S6).^39^ As expected, the maximum signal of the NSE occurs when τ_1,RF_ = τ_2,RF_ = τ_RF_ and the intensity of the echo signal decreases with increasing τ_RF_ (Figure 4A). This decay reflects the transverse nuclear spin relaxation. This decay is further illustrated by the gray dashed trace in the figure, which shows a clear decrease with increasing τ_1,RF_. Figure 4B presents the NSE decay measured at three different temperatures.

**Figure 4:**
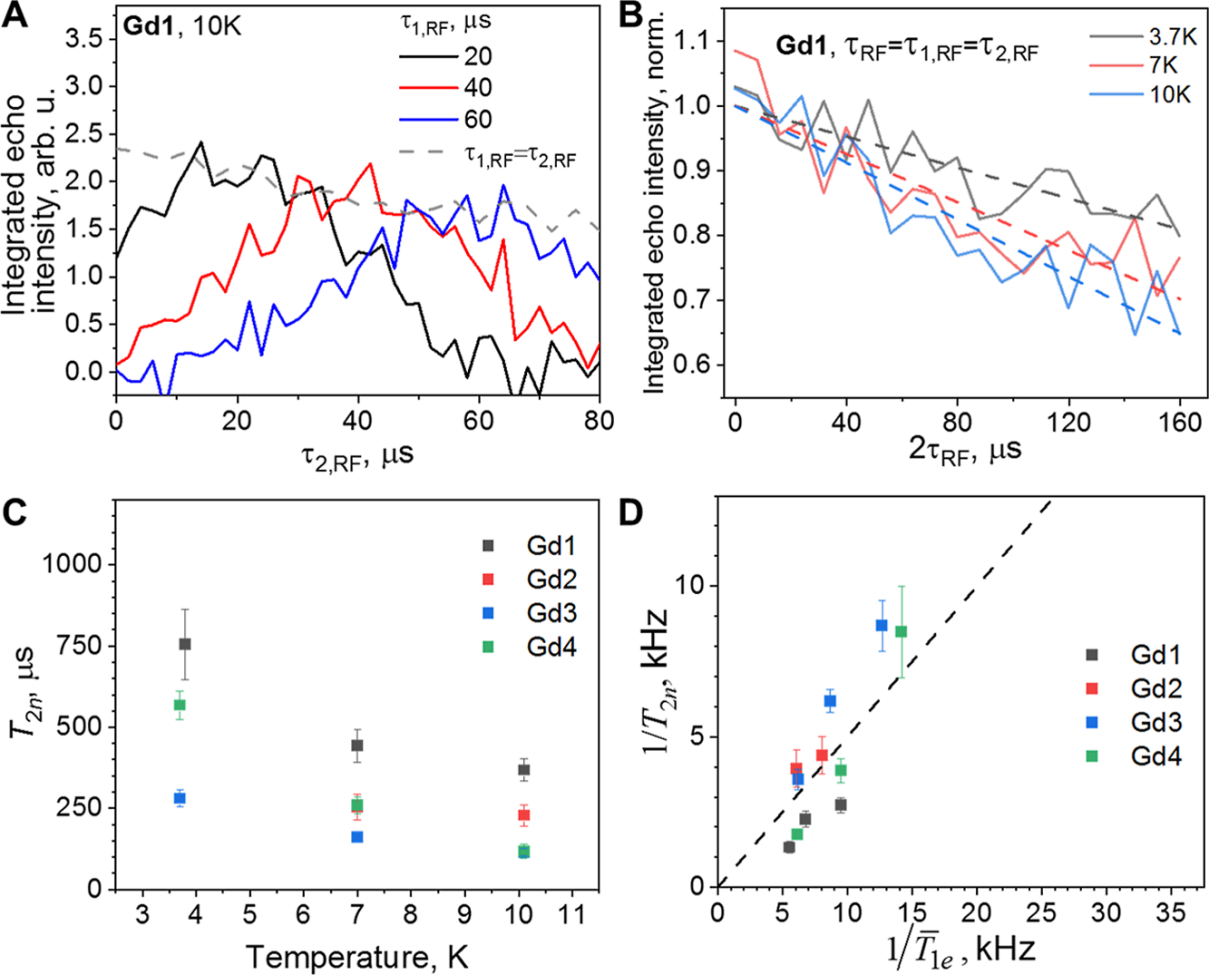
(A) ^19^F NSE of **Gd1** recorded at 10 K, with RF delays τ_1,RF_ of 20, 40, and 60 μs (solid lines), and the corresponding nuclear spin echo decay trace recorded with τ_1,RF_ = τ_2,RF_ (dashed gray line). (B) NSE decays of ^19^F in **Gd1** recorded at 3.7 K, 7 K, and 10 K. The data for the other complexes are shown in Figure S7. (C) *T*_2n_ as a function of temperature for **Gd1–Gd4**. (D) 1/*T*_2n_ measured at different temperatures and plotted as a function of $1/{\mathrm{T̅}}_{1e}$ measured by saturation recovery for **Gd1–Gd4**. The dashed line corresponds to ${T_{2n}}^{-1}={\left(2{\mathrm{T̅}}_{1e}\right)}^{-1}$ . The NSE decays were measured at ν_RF_ – ν_I_ = 36 kHz (**Gd1**), −42 kHz (**Gd2**), 40 kHz (**Gd3**), 20 kHz (**Gd4**), and the corresponding frequency positions are marked as blue arrows in Figure 3A.

The longest τ_1,2,RF_ that can be used in such measurements is determined by the longest mixing period *T* in the Mims ENDOR sequence that can provide reasonable signal-to-noise ratio (SNR) and is limited primarily by *T*_1e_.^40^ The shortest τ_1,2,RF_ is zero (note that we defined τ_1,2,RF_ between the edges of the adjacent pulses (cf. Figure 2B) and not between the centers of the pulses). *T*_2n_ was determined by fitting the NSE intensity *I*_echo_(τ_*RF*_) to

10 $$I_{echo}\left(\tau_{RF}\right)\propto \exp\left[-\frac{2\tau_{RF}}{T_{2n}}\right]$$

Note that SNR restrictions allow recording only the early regions of the decay curves (maximum τ_RF_ between 80 and 200 μs for different experiments), and in the data analysis we assumed a monoexponential decay. The phase cycle on the RF pulse ensured that the echo decay reaches zero at sufficiently large τ_RF_. Therefore, the initial linear region of the NSE decay curves, as observed in Figure 4B, allows determining *T*_2n_ (dashed lines in Figure 4B show fitting for **Gd1**, data for **Gd1**–**Gd4** are compared in Figure S7).

The temperature dependence of *T*_2n_ for **Gd1–Gd4**, presented in Figure 4C, shows that for Gd­(III) complexes with DO3A chelates (**Gd1** and **Gd2**) 1/*T*_2n_ is on the order of 1–4 kHz. For proteins labeled with this Gd-DO3A, the overall ^19^F line width is on the order of 15–25 kHz.^11^ Thus, the intrinsic line width, determined by 1/*T*_2n_ is overridden by the other broadening mechanisms,^19^ among which the distance distribution is the dominant.^11^ For Gd­(III) in PyMTA complexes (**Gd3** and **Gd4**), the relaxation rate is estimated to be 5–8 kHz, thus contributing somewhat more significantly to the line width than **Gd1** and **Gd2**, but it is still small. For comparison, the broadening due to dipolar interaction of ^19^F nuclei with adjacent ^1^H and ^19^F nuclei was estimated to be on the order of 6–11 kHz, and the broadening due to the chemical shift anisotropy at W-band to be between 5 and 7 kHz.^11^ We therefore conclude that the ^19^F nuclear decoherence does not appear to be the factor limiting ^19^F-ENDOR spectral resolution in Gd­(III) labeled proteins. In case that this would have been the major contribution to the line width, the largest distance that can be measured by the splitting of the ENDOR doublet is 21–25 Å at 10 K. Nevertheless, this will be associated with a large decrease in SNR because of the *r*_GdF_^**–**6^ dependence of the ENDOR efficiency^41^ and the requirement for a long τ for measuring long distances.

Next, we address the decoherence mechanism. The temperature dependence of *T*_2n_ reflects its dependence on ${\mathrm{T̅}}_{1e}$ , the values of which are given in Table S3. The values of $1/{\mathrm{T̅}}_{1e}$ for **Gd1–Gd4** range from 7 to 22 kHz, depending on the temperature and the compound, whereas the *a*_⊥_ values are 30 to 70 kHz, thus $a_{⊥}>1/{\mathrm{T̅}}_{1e}$ . With the experimental *a*_⊥_ and $1/{\mathrm{T̅}}_{1e}$ values, eq 1 predicts *T*_2n_ values of 4–20 ns for **Gd1**–**Gd3** and 30–70 ns for **Gd4**, which is many orders of magnitude faster than the experimentally obtained values (100–800 μs). As mentioned earlier, this discrepancy is expected, because eq 1 is valid under the condition of *a*_⊥_ ≪ 1/*T*_1e_. Next, we considered the agreement with eq 2, which applies to the other extreme.

In Figure 4D we present 1/*T*_2n_ as a function of $1/{\mathrm{T̅}}_{1e}$ . It demonstrates a good agreement with eq 2, which is shown as a dashed line in this panel. This shows that the ^19^F nuclear decoherence is determined primarily by the electron spin–lattice relaxation. The use of $1/{\mathrm{T̅}}_{1e}$ measured by inversion recovery provided poorer agreement with eq 2, owing to the overestimation of $1/{\mathrm{T̅}}_{1e}$ compared to the values determined by saturation recovery (see Figure S8). Furthermore, using the averaged values $1/{\mathrm{T̅}}_{1e}$ (taken as first moment of the relaxation function, cf. eq 4) provides better agreement with the theory than using nonaveraged values 1/*T*_1e_, because it takes into account the distributions in spin–lattice relaxation rates as manifested in the values of β. Figure S8 also shows the linear regression of the experimental data, where a slopes in the range of 0.33–0.54 were obtained, compared to a slope of 0.5 predicted by eq 2. We also looked for the dependence of 1/*T*_2n_ on the Gd–F distance. We compared **Gd3** and **Gd4,** which have Gd–F distances of 10 and 13 Å, respectively, and very similar ${\mathrm{T̅}}_{1e}$ and β values (see Table S3). Focusing on Figure 4C, we observe that *T*_2n_ for **Gd4** is longer, but the difference decreases with temperature, and at 10 K they become similar. We thus conclude that for the samples measured, we do not see an unambiguous dependence on the distance.

Equation 2 was derived for the case of *S* = 1/2 and by using it we imply that our system behaves as an effective *S* = 1/2. This is justified by the fact that the majority of the excited spins are attributed to CT and by the independence of ${\mathrm{T̅}}_{1e}$ on the field within the EPR spectrum, and thus on *m*_S_.^42^ The ENDOR spectra recorded at 3.7 K at the CT, are still dominated by the ENDOR spectrum corresponding to the CT (see Figure S3), supporting the effective *S* = 1/2 behavior. Nevertheless, the possibility of spin flips with |Δ*m*_S_| > 1 are not considered, and their rate can be different from the measured $1/{\mathrm{T̅}}_{1e}$ . The existence of such flips for Gd­(III) with *S* = 7/2 was reported in Gd­(III)–Gd­(III) RIDME (relaxation induced dipolar modulation enhancement) measurements.^43^ The relatively good agreement with the prediction of eq 2 suggests that these effects are minor.

**Gd1**, **Gd3**, and **Gd4** have only one ^19^F nucleus per molecule, and therefore, we do not expect ^19^F spin–spin contributions to nuclear decoherence. **Gd2** has three ^19^F nuclei in close vicinity, and its *T*_2n_ is somewhat smaller than that of **Gd1**, which may hint at such a contribution. To explore the potential effect of nuclear spin–spin interactions on nuclear decoherence in these molecules, we measured the ^1^H *T*_2n_.

### ^1^H Relaxation Measurements

The ^1^H ENDOR spectra of **Gd1–Gd4** comprise several doublets (Figure 3B), corresponding to different protons in the complex. The spectra can be successfully simulated, as we showed previously,^10^ and the resolved doublets in the Mims ENDOR spectra measured with τ = 300–600 ns correspond to Gd–H distances in the range of 2.7–5.5 Å. In the spectra recorded with τ = 2000 ns these resolved doublets are masked because of the Mims ENDOR blind spots, and the central region shows the enhanced contributions of the weakly coupled protons, from the complex itself and possibly from residual protons in the solvent (Figure 3B).

^1^H NSE decays of **Gd1** and **Gd3** are shown in Figures 5A and S9 (the nuclear spin echoes are presented in Figure S10). Because of the much higher ENDOR efficiency (due to the large number of protons), the SNR of these traces is significantly better than those of the ^19^F traces. The decays are substantially nonexponential and reveal two exponential components; this is particularly clear for the ^1^H NSE decay of **Gd1** at 3.7 K (Figure 5A). Following an initial fast decay, the relaxation becomes very slow. While the rates of the fast component (50–80 kHz) are significantly higher than those of the ^19^F nucleus of **Gd1** (1–3 kHz), those of the slow component (0.3–1 kHz) are lower than the ^19^F ones.

**Figure 5:**
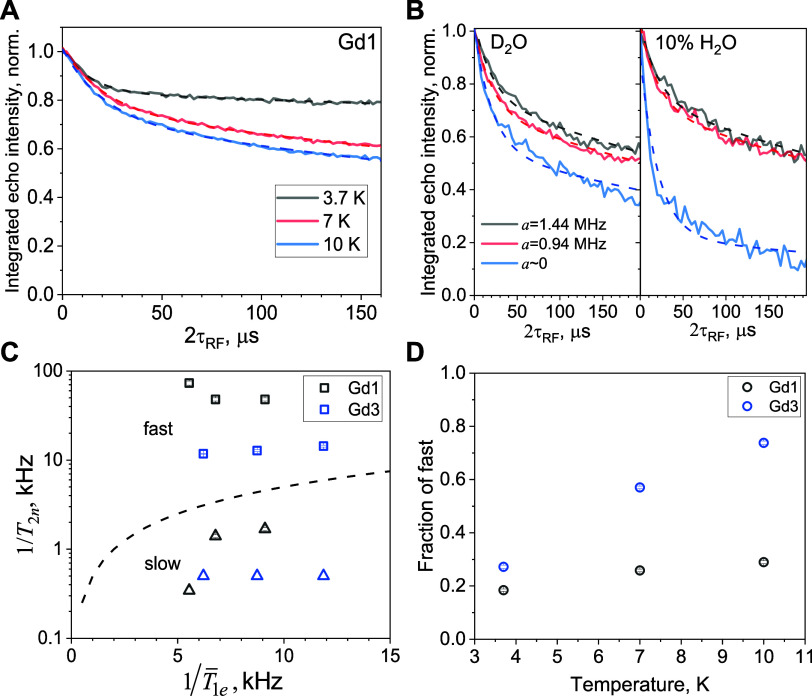
(A) NSE decays of ^1^H in **Gd1** recorded at ν_RF_ = ν_I_ – 0.47 MHz and 3.7–10 K. Solid lines—experimental data, dashed lines—biexponential fits (parameters are listed in Table S4). (B) NSE decay curves measured for ^1^H at 10 K for **Gd1** dissolved in purely deuterated solvent, 3:1 D_2_O:glycerol-d_8_ (left panel) and with the addition of 10 vol % H_2_O. Measurements were performed at the ν_RF_ = ν_I_ – 0.72 MHz and τ = 350 ns (black lines), ν_RF_ = ν_I_ – 0.47 MHz and τ = 600 ns (red lines), ν_RF_ ≈ ν_I_ and τ = 2 μs (blue lines). The corresponding values of the hyperfine splitting are shown in the legend, and the frequency positions are marked with blue arrows in Figure 3B. Dashed lines are the biexponential fits using the same relaxation times for fast and slow relaxation components (10 K) as obtained from fitting the data in panel (A) (see Table S4). (C) The 1/*T*_2n_ value of the fast and slow components of the ^1^H NSE decays of **Gd1** and **Gd3** as a function of $1/{\mathrm{T̅}}_{1e}$ , measured by saturation recovery technique. The dashed line corresponds to $1/T_{2n}=1/2{\mathrm{T̅}}_{1e}$ . (D) The relative contribution of the fast component of the NSE decay as a function of temperature.

To understand the source of the two contributions, we carried out measurements as a function of the hyperfine couplings. These are shown in left panel of Figure 5B, for hyperfine constants *a* = 1.44, 0.94, and ∼0 MHz. We see that for *a* ∼ 0 MHz the relative contribution of the fast component is larger than that of the other two. This could be a consequence of the contribution of the energy-conserving nuclear flip-flops driven by dipolar interaction between protons, which is expected to be more effective between very weakly coupled protons that have similar resonance frequencies. To substantiate this assignment, we prepared **Gd1** in a solution containing 10 vol % H_2_O (the concentrations of **Gd1** and glycerol-d_8_ were kept the same). The ^1^H ENDOR spectra (Figure S11) of this sample corroborate the increased contributions of weakly coupled protons in spectra acquired with long τ values. As seen from the ^1^H NSE decay measurements (Figure 5B, right panel), for the protons with *a* ∼ 0 there is a significant increase in the fast component upon the addition of protons to the solution. In contrast, the curves corresponding to *a* = 1.44 and 0.94 MHz were barely altered. Moreover, the slowly relaxing component seems to be characterized by a similar decay rate for all *a* values, independent of the H_2_O content. In fact, all decay curves presented in Figure 5B could be roughly simulated by biexponential curves with the same decay times as obtained for **Gd1** at 10 K and *a* = 0.94 MHz, while only varying the relative fractions of fast and slow components. The fit parameters are listed in Table S5. This indicates that the protonation of the matrix influences the relative amount of the two populations rather than changing the relaxation rates of each population. These measurements substantiate the assignment of the fast component to protons experiencing flip-flops with nearby protons having similar frequencies, i.e., nuclear spin diffusion.

The dependence of the fast and slow components of the ^1^H NSE relaxation on $1/{\mathrm{T̅}}_{1e}$ for **Gd1** and **Gd3** (*a* = 0.94 MHz) is shown in Figure 5C. The dependence is quite weak, which is expected for the fast component that is driven by dipolar flip-flop, and indeed the rates are significantly higher than expected by the limit set by eq 2. For the slow component, unexpectedly, *T*_2n_ ≫ 2*T*_1e_, which violates eq 2. Finally, the plot of the relative contribution of the fast component as a function of temperature is shown in Figure 5D. While for **Gd1** the dependence is mild, for **Gd3** it is quite strong and unexpected. Currently we do not understand this behavior.

## Conclusions

To conclude, our measurements of ^19^F nuclear phase memory times for a series of Gd­(III) model complexes, differing by chelate type and Gd–F distance, show that the nuclear decoherence is controlled by the electron *T*_1e_ relaxation as predicted by eq 2. The contribution of the intrinsic nuclear transverse relaxation to the commonly observed line widths of ^19^F ENDOR with the Gd­(III) spin labels used is negligible compared to other broadening mechanisms, like distance distributions, and therefore is not a determinant of the resolution. The ^1^H NSE decays show more complicated behavior, apparently due to nuclear–nuclear interactions, given the many protons present. At present, however, we do not have a complete picture of the proton relaxation in the studied complexes, and further experimental and theoretical efforts are needed to clarify the underlying mechanisms. The reported results may be of interest beyond the scope ENDOR spectroscopy, particularly in contexts where electron and nuclear spin relaxation interplay is significant, such as paramagnetic relaxation enhancement (PRE), and dynamic nuclear polarization (DNP) in the solid state.

## Supplementary Material



## Notes

Details of synthesis and sample characterization, ^19^F-ENDOR spectra recorded at different temperatures, ^1^H ENDOR spectra recorded with various solvent protonation degrees, ENDOR spectra simulation details and parameters, NSEs recorded without RF phase cycling, ^19^F and ^1^H NSE decay curves, data on electron spin–lattice relaxation (PDF)
