# Supplementary material for: Decoherence of Hyperfine Coupled 19F and 1H Nuclei in Gadolinium(III) Model Complexes
Source: J Phys Chem B. 2025 Jun 21;129(26):6604–12. doi: 10.1021/acs.jpcb.5c03224 (PMC12235618; doi:10.1021/acs.jpcb.5c03224)
Supplement: Supplementary file 1 [file jp5c03224_si_001.pdf]

Supporting information

Decoherence of Hyperfine Coupled ^{19}F and ^{1}H Nuclei in Gadolinium(III) Model Complexes

Alexey Bogdanov,[a,] Veronica Frydman,[b] Xun-Cheng Su,[c] and Daniella Goldfarb[a,*]*

[a] Department of Chemical and Biological Physics,
The Weizmann Institute of Science, P. O. Box 26, Rehovot 7610001 Israel
[b] Department of Chemical Research Support,
The Weizmann Institute of Science, P. O. Box 26, Rehovot 7610001 Israel
[c] State Key Laboratory of Elemento-Organic Chemistry,
Nankai University, Tianjin 300071 P. R. China.
* alexey.bogdanov@weizmann.ac.il, * daniella.goldfarb@weizmann.ac.il

Contents

S1. Synthesis and characterization details S2
S2. Examples of T_{1e} measurements S6
S3. ENDOR spectra and simulations S7
S3. RF phase cycling in the nuclear spin echo experiment........ S10
S4. ^{19}F NSE decays and exponential fitting........ S11
S5. Electron spin-lattice relaxation of Gd1-Gd4........ S12
S6. Dependence of $1/T_{2n}$ on $1/T_{1e}$ measured using different techniques S13
S7. ^{1}H nuclear spin echo decay in Gd1 and Gd3 .... S14
S8. ^{1}H ENDOR spectra and NSE decays of Gd1 in deuterated and partially protonated solvent........ S16
Supplementary references........ S17

S1. Synthesis and characterization details

*Synthesis of **Gd4**.*

Complex **Gd4** was produced using the synthetic strategy outlined in **Scheme S1**. The details of the synthetic procedures and analysis results are listed below.

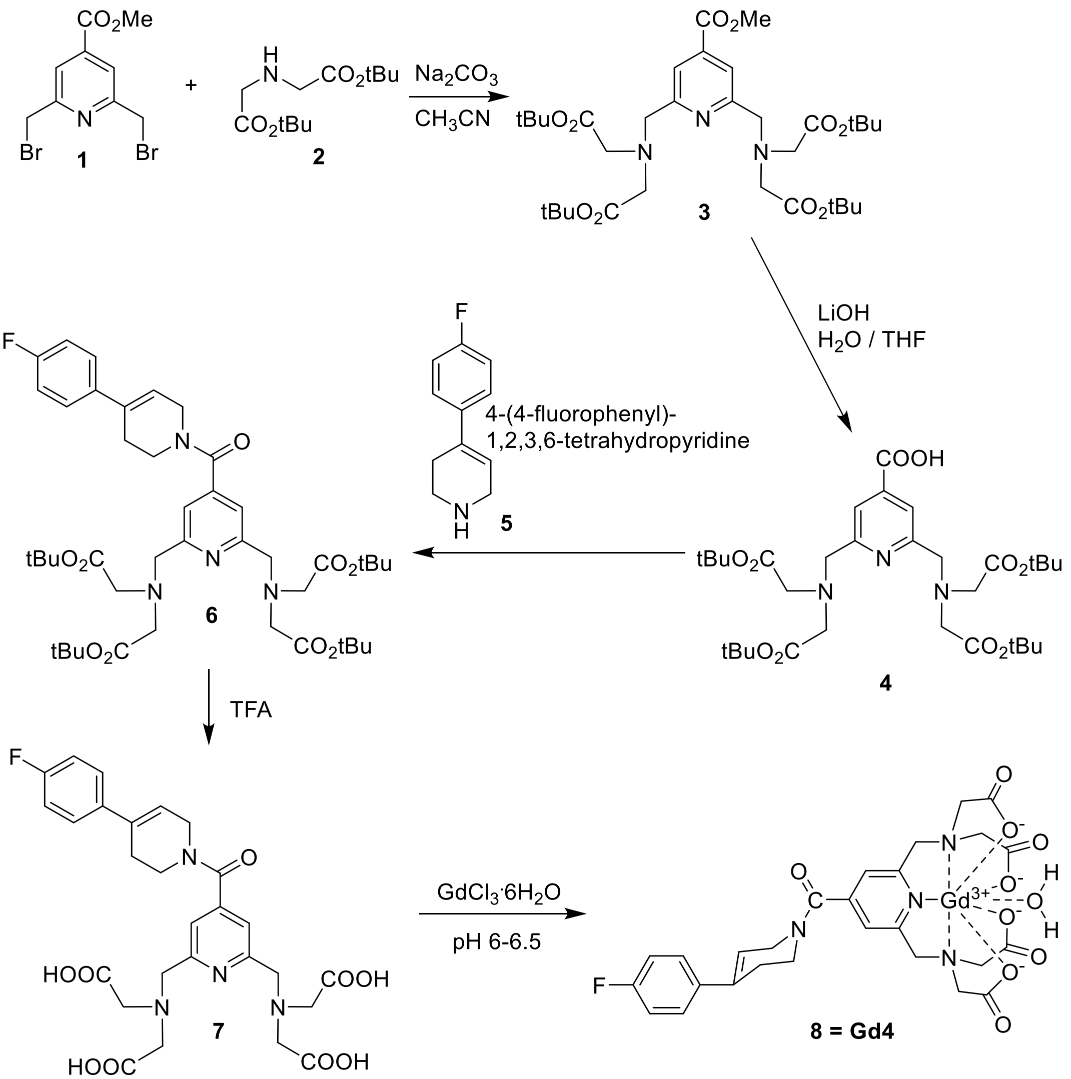

Scheme S1. General scheme of synthesis of **Gd4**.

General Procedures

Column chromatography was carried out on neutral alumina deactivated to the desired degree by addition of the required amount of water to neutral alumina grade I. ^{1}H NMR spectra were recorded on a Bruker Avance 400 spectrometer and chemical shifts are given in ppm referenced to the solvent peak. Mass spectra were recorded as ESI spectra.

Tetra(*tert*-butyl)2,2′,2″,2‴[4-(methoxycarbonyl)pyridine-2,6-diyl]bis(methylenenitrilo)-tetrakisacetate (3)

Compound **3** was prepared from 2,6-bis(bromomethyl)pyridine-4-carboxylic acid methyl ester (**1**) and di-*tert*-butyl-iminodiacetate (**2**) following the procedure described in the literature.[S3] ^{1}H NMR (400 MHz, $CDCl_3$) δ 1.48 (s, 36H), 3.51 (s, 8H), 3.94 (s, 3H), 4.11 (s, 4H), 8.03 (s, 2H).

Tetra(*tert*-butyl)2,2′,2″,2‴-[(4-carboxy)pyridine-2,6-diyl)bis(methylenenitrilo)]-tetrakis acetate (4)

The pentaester **3** (1.1 g, 1.69 mmol) was dissolved in THF (10.7 mL) and a solution of LiOH (44 mg, 1.84 mmol) in H_2O (1.8 mL) was added. The mixture was stirred at room temperature overnight. The THF was evaporated at reduced pressure, the residue was diluted with H_2O (10.2 mL) and the pH was adjusted to 4.5 while cooling in an ice-water bath, by addition of a 1M HCl solution. The mixture was extracted with DCM and the organic extracts were washed with brine and dried over Na_2SO_4. Evaporation of the solvent under reduced pressure afforded compound **4** as a colorless oil (0.85 g, 85%). ^{1}H NMR (400 MHz, $CDCl_3$) δ 1.48 (s, 36H), 3.53 (s, 8H), 4.13 (s, 4H), 8.11 (s, 2H).

Tetra-tert-butyl-2,2',2'',2'''-(((4-(4-(4-fluorophenyl)-1,2,3,6-tetrahydropyridine-1-carbonyl)pyridine-2,6-diyl)bis(methylene))bis(azanetriyl))tetraacetate (6)

The acid **4** (0.44 g, 0.691 mmol) was dissolved in anhydrous DCM (8 mL) under nitrogen. To this solution, DIPEA (0.447 g, 3.4 mmol) was added, followed by PyBOP (0.576 g, 1.1 mmol). The mixture was stirred for 5 minutes and 4-(4-fluorophenyl)-1,2,3,6-tetrahydropyridine (**5**) (0.159 g, 0.9 mmol) was added. The mixture was stirred at room temperature under nitrogen overnight and then it was diluted with DCM (30 mL), washed with water (2 x 10 mL) followed by brine (1 x 10 mL) and dried over Na_2SO_4. The solvent was evaporated and the residue was purified by column chromatography on neutral alumina grade II using DCM/MeOH 1% as eluent. Since only a fraction of the material eluted pure, the impure fractions were collected and purified twice more by column

chromatography on neutral alumina grade II using DCM/MeOH 0.5% as eluent. The final product **6** was obtained as a colorless oil (0.194 g, 35%). ^{1}H NMR (400 MHz, $CDCl_3$) δ 1.47 (s, 36H), 2.63 (m, 2H) 3.51 (s, 10H), 3.61 (br t, 1H), 3.99 (br t, 1H), 4.07 (s, 4H), 6.09 (br m, 1H), 7.04 (m, 2H), 7.38 (m, 2H), 7.65 (m, 2H).

2,2',2'',2'''-(((4-(4-(4-fluorophenyl)-1,2,3,6-tetrahydropyridine-1-carbonyl)pyridine-2,6-diyl)bis(methylene))bis(azanetriyl))tetraacetic acid (7)

The tetraester **6** (0.18 g, 0.226 mmol) was dissolved in anhydrous DCM (6 mL) and TFA (3 mL) was added. The mixture was sealed and stirred overnight at room temperature in the dark. The solvents were evaporated at reduced pressure and the residue was resuspended in anhydrous DCM (10 mL) and evaporated to remove the remaining TFA (three times). The solid residue was washed with anhydrous ethyl ether, followed by anhydrous DCM, and dried under vacuum, affording the tetraacid **7** in quantitative yield. ^{1}H NMR (400 MHz, MeOD-d_4) δ 2.5 (m, 2H), 3.4 (t, 2H), 3.57 (br s, 10H), 3.90 (br s, 4H), 6.05 (m, 1H), 6.97 (m, 2H), 7.34 (m, 2H), 7.75 (m, 2H).

Gd(III) chelate (8)

The tetraacid **7** (70.6 mg, 0.123 mmol) was suspended in H_2O (1 mL), and $GdCl_3{\cdot}6H_2O$ (51 mg, 0.136 mmol) was added. The pH was adjusted to 6-6.5 with 0.25M NaOH. The mixture was stirred at room temperature, and the pH was maintained at a value between 6 and 6.5 by the addition of small aliquots of 0.25M NaOH. Once the pH remained stable in that range, the stirring was continued overnight at room temperature. The precipitate was collected by centrifugation (6600 rpm, 4 min, 4 °C). The pellet was washed with water (1 mL) and collected by centrifugation under the same conditions as before. The washing was repeated once more. The pellet was dried under vacuum, affording the chelate **8** as a white solid (75 mg, 83%). MS (ESI^+): m/z 750.08 ($[M+Na]^+$), 772.06 ($[M-H+2Na]^+$) Elemental Composition Analysis (isotope fit): $C_{27}H_{26}FGdN_4O_9$.

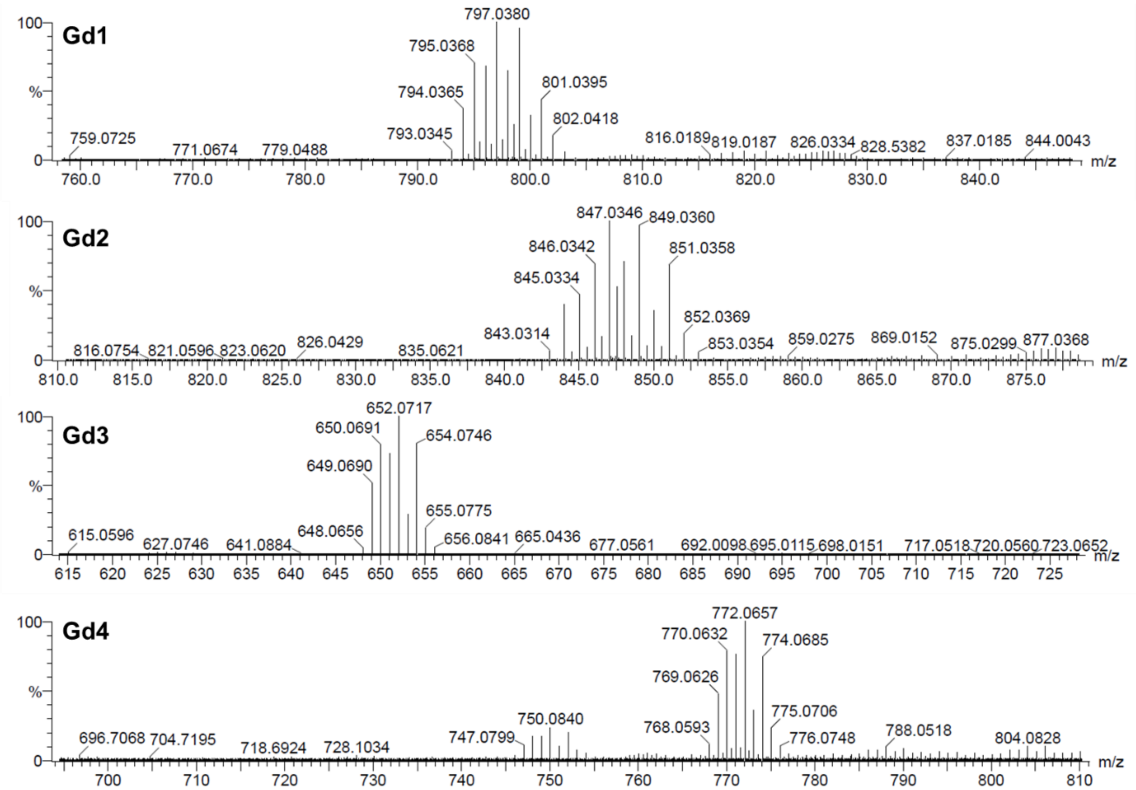

Figure S1. High resolution mass spectra of **Gd1–Gd4**; the found m/z and elemental composition analyses (ECA, isotope fit) are presented below. For **Gd1**: MS (ESI$^+$) m/z 797.04 ([M+H]$^+$), ECA: $C_{26}H_{30}BrFGdN_5O_6S$. For **Gd2**: MS (ESI$^+$) m/z 847.03 ([M+H]$^+$), ECA: $C_{27}H_{30}BrF_3GdN_5O_6S$. For **Gd3**: MS (ESI$^-$): m/z 652.07 ([M–H]$^-$), ECA: $C_{21}H_{24}FGdN_4O_9$. For **Gd4**: MS (ESI$^+$): m/z 750.08 ([M+Na]$^+$), 772.06 ([M–H+2Na]$^+$) ECA: $C_{27}H_{26}FGdN_4O_9$.

S2. Examples of T_{1e} measurements

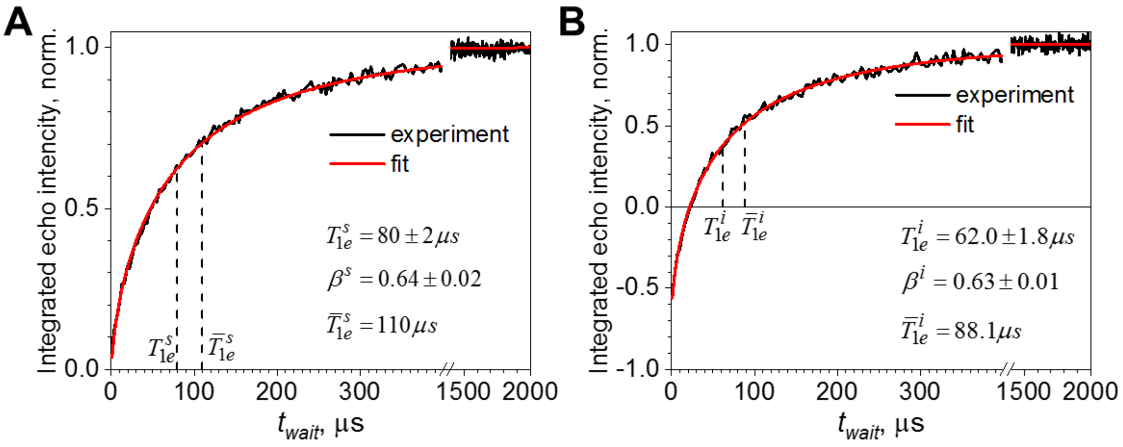

Figure S2. Saturation recovery (A) and inversion recovery (B) traces of **Gd1** at 10 K and their stretched exponential fitting. Fitting parameters and corresponding relaxation times are listed and marked in the figure.

S3. ENDOR spectra and simulations

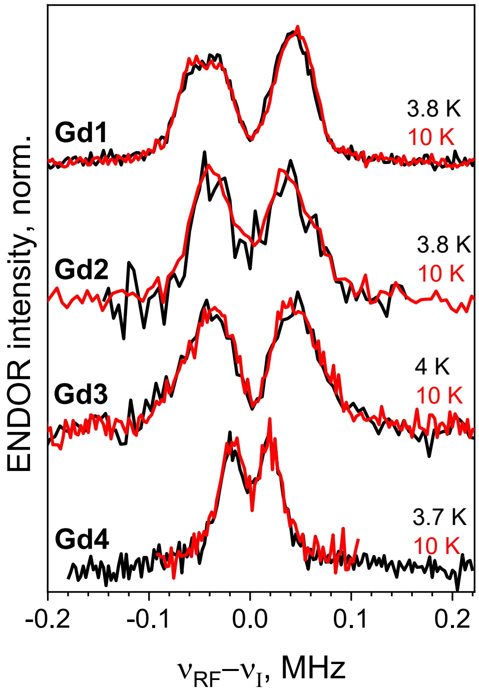

Figure S3. Experimental ^{19}F-ENDOR spectra of **Gd1–Gd4,** recorded at temperatures 3.7–4 K and 10 K with τ=2 μs (the spectrum of **Gd1** at 3.8 K was recorded with τ=3 μs). The temperatures are specified in the figure.

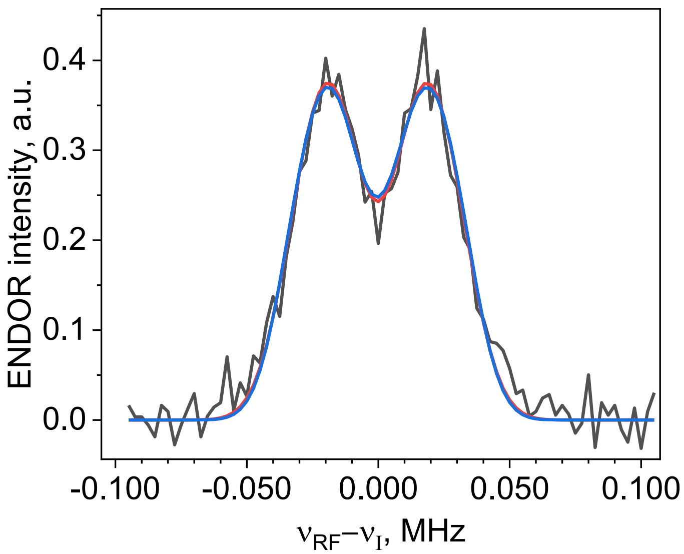

Figure S4. Experimental ^{19}F ENDOR spectrum of **Gd4** (black line) and its simulation without taking into account the CS anisotropy (red line) , and with $(\delta_{xx}, \delta_{yy}, \delta_{zz}) = (98, -29, -69)$, as used for **Gd1** (blue line). The orientation of Gd–F vector in the CS principal frame is given by $(\gamma, \rho) = (39°, 0°)$. The value of $|a_\perp|$ obtained from both simulations is 31.6 kHz, and the Gaussian linewidths are 21.3 kHz and 18.9 kHz, respectively.

Table S1. Simulation best fit parameters for ^{19}F-ENDOR spectra of the studied Gd complexes

complex	$\lvert a_{\perp} \rvert$, kHz	r_{GdF}, Å	Δ_G, kHz	Δ_L, kHz	ν_I, MHz	CS tensor, ppm	γ, ρ, deg.
**Gd1**	72.6±0.6	10.1±0.1	25.8±1.0	0	136.42 ($\nu_{I,0}$)	98, –29, –69	57±10, 75±9
**Gd2**	64.0±0.9	10.5±0.1	24±4	7±3	136.35	–	–
**Gd3**	73.7±0.3	10.03±0.05	0	20±1	136.40	–	–
**Gd4**	31.6±0.8	13.3±0.3	21±2	0	136.38	–	–

Table S2. Simulation best fit parameters for ^{1}H-ENDOR spectra of the studied Gd complexes. The assignment of the protons is given in **Fig. S5**.

complex		relative contribution[a]	$\lvert a_{\perp} \rvert$, MHz	r_{GdH}, Å	Δ_G, kHz	Δ_L, kHz	ν_I, MHz
**Gd1**	H1	1.00	0.95±0.01	4.36±0.05	2±2	32±2	144.97
	H2	1.28±0.02	1.53±0.01	3.73±0.10	98±2	8±2	
	H3	0.86±0.03	0.52±0.01[b]	5.35±0.10[b]	56±10	177±10	
	H4	1.11±0.05	0.094±0.005[b]	9.4±0.5[b]	116±6	0	
**Gd3**	H1	1.00	0.955±0.005	4.36±0.02	58±2	0	144.94
	H2	1.14±0.17	1.66±0.01	3.62±0.02	0	87±8	
	H3	0.74±0.11	1.40±0.01	3.84±0.03	0	60±8	
	H4	0.23±0.05	0.47±0.01	5.51±0.11	24±2	0	
	H5	0.22±0.04	2.71±0.05	3.08±0.06	133±20	0	
	H6	3.88±0.15	0.33±0.04[b]	6.2±0.8[b]	300±10	54±10	

[a] The relative amount of protons with the corresponding $a_{\perp}$, derived from the relative integral intensity in the Mims ENDOR spectrum.

[b] Weakly coupled protons of the complex or matrix

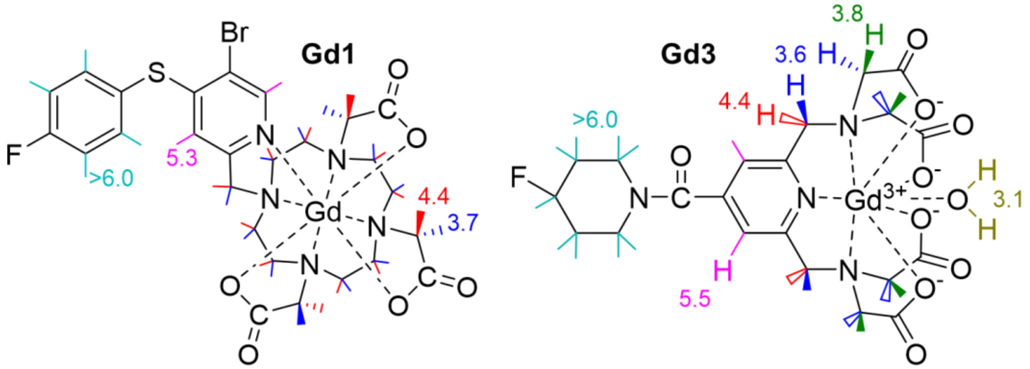

Figure S5. The structure of **Gd1** and **Gd3,** with the tentative assignment of different protons listed in **Table S2**. The figures next to the protons correspond to Gd–H distances in angstrom, and the color codes correspond to those used in **Table S2**.

S3. RF phase cycling in the nuclear spin echo experiment

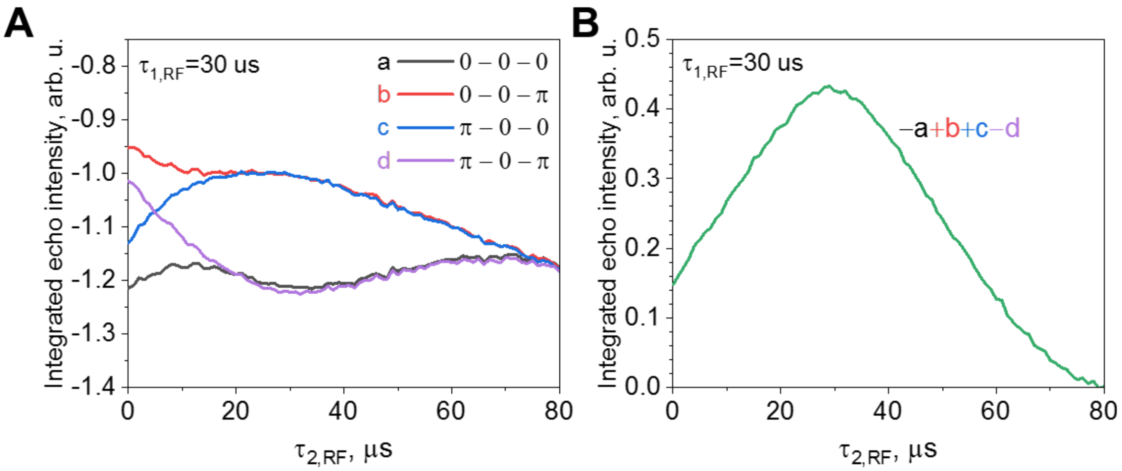

Figure S6. (**A**) ^{1}H NSE traces of **Gd1** at 10 K with $\tau_{RF,1}$=30 μs, recorded at $\nu_{RF}=\nu_I-0.47$ MHz, shown for the individual phase-cycle steps a, b, c, d (the relative phases of the three RF pulses in NSE sequence, are given in the legend). (**B**) A superposition of the traces shown in panel (**A**) that produces the clean NSE.

S4. ^{19}F NSE decays and exponential fitting

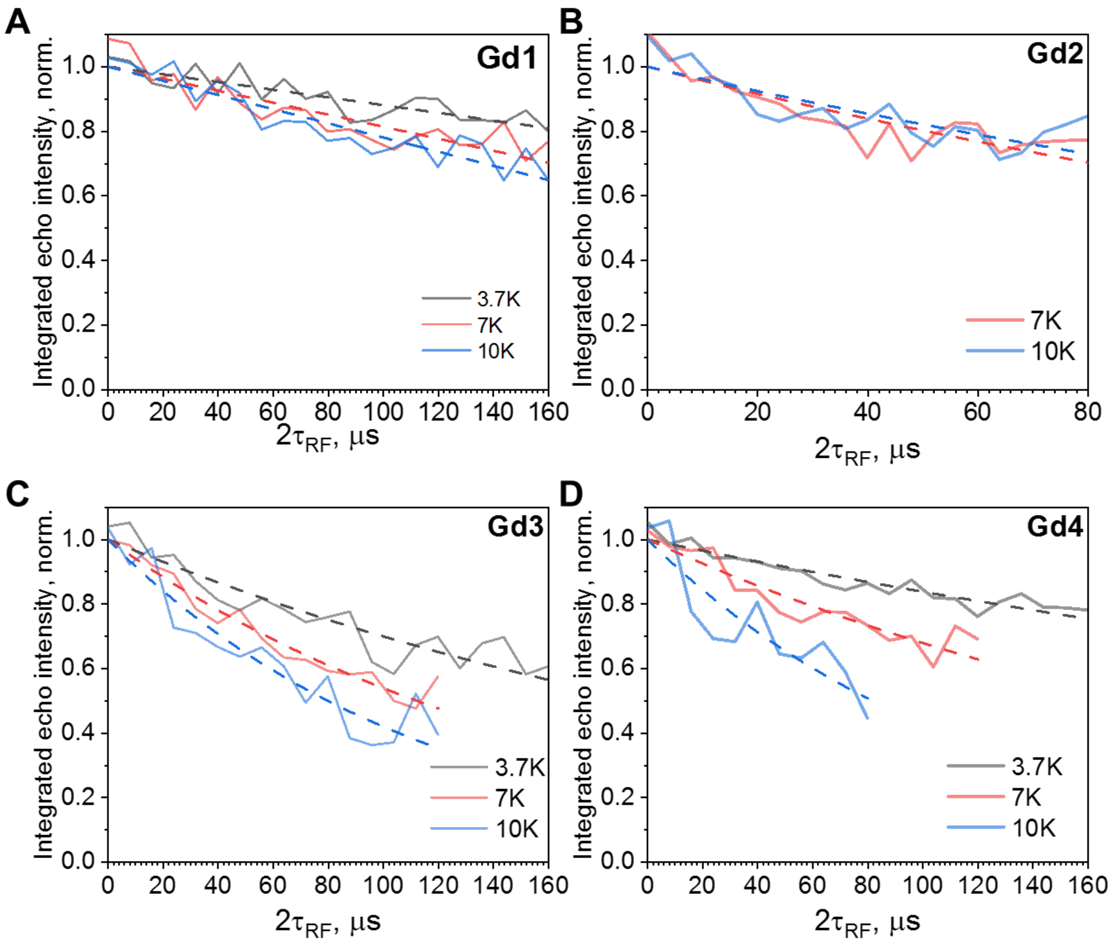

Figure S7. NSE decays of ^{19}F in **Gd1** (**A**), **Gd2 (B), Gd3 (C), and Gd4 (D),** recorded at temperatures 3.7 –10 K. The NSE decays were measured at $\nu_{RF} - \nu_I$ = 36 kHz (**Gd1**), –42 kHz (**Gd2**), 40 kHz (**Gd3**), 20 kHz (**Gd4**), and the corresponding frequency positions are marked as blue arrows in **Fig. 3A** *(main text)*.

S5. Electron spin-lattice relaxation of Gd1-Gd4

Table S3. The fit parameters of the inversion and saturation recovery experiments for **Gd1-Gd4** at 3.7–10 K. The parameters are defined in the main text (eqs. (3)-(4)). Characteristic experimental errors are shown in the table headers for experimentally determined $T_{1e}^{i,s}$ and $\beta^{i,s}$.

complex	Temperature, K	Inversion recovery				Saturation recovery			
		T_{1e}^{i}, μs ±1.5 μs	β^{i} ±0.01	$\bar{T}_{1e}^{i}$, μs	$1/\bar{T}_{1e}^{i}$, kHz	T_{1e}^{s}, μs ±2 μs	β^{s} ±0.02	$\bar{T}_{1e}^{s}$, μs	$1/\bar{T}_{1e}^{s}$, kHz
**Gd1**	3.7	142.6	0.65	195	7.01	154	0.76	180	5.54
	7	90.0	0.64	125	11.11	101	0.61	148	6.78
	10.1	62.0	0.63	88.1	16.59	80	0.64	110	9.11
**Gd2**	3.7	130.0	0.68	169	7.69	152	0.69	195	5.13
	7	91.6	0.66	123	10.92	130	0.70	164	6.09
	10.1	57.0	0.61	84.0	17.53	104	0.75	124	8.05
**Gd3**	3.7	119.2	0.68	155	8.39	122	0.68	161	6.21
	7	76.5	0.65	104	13.07	93	0.72	114	8.74
	10.1	47.7	0.64	66.4	20.95	68	0.71	84	11.9
**Gd4**	3.7	126.6	0.72	157	7.90	130	0.71	162	6.16
	7	71.1	0.66	95.0	14.06	80	0.67	105	9.49
	10.1	43.7	0.64	60.4	22.89	54	0.68	71	14.2

S6. Dependence of $1/T_{2n}$ on $1/T_{1e}$ measured using different techniques

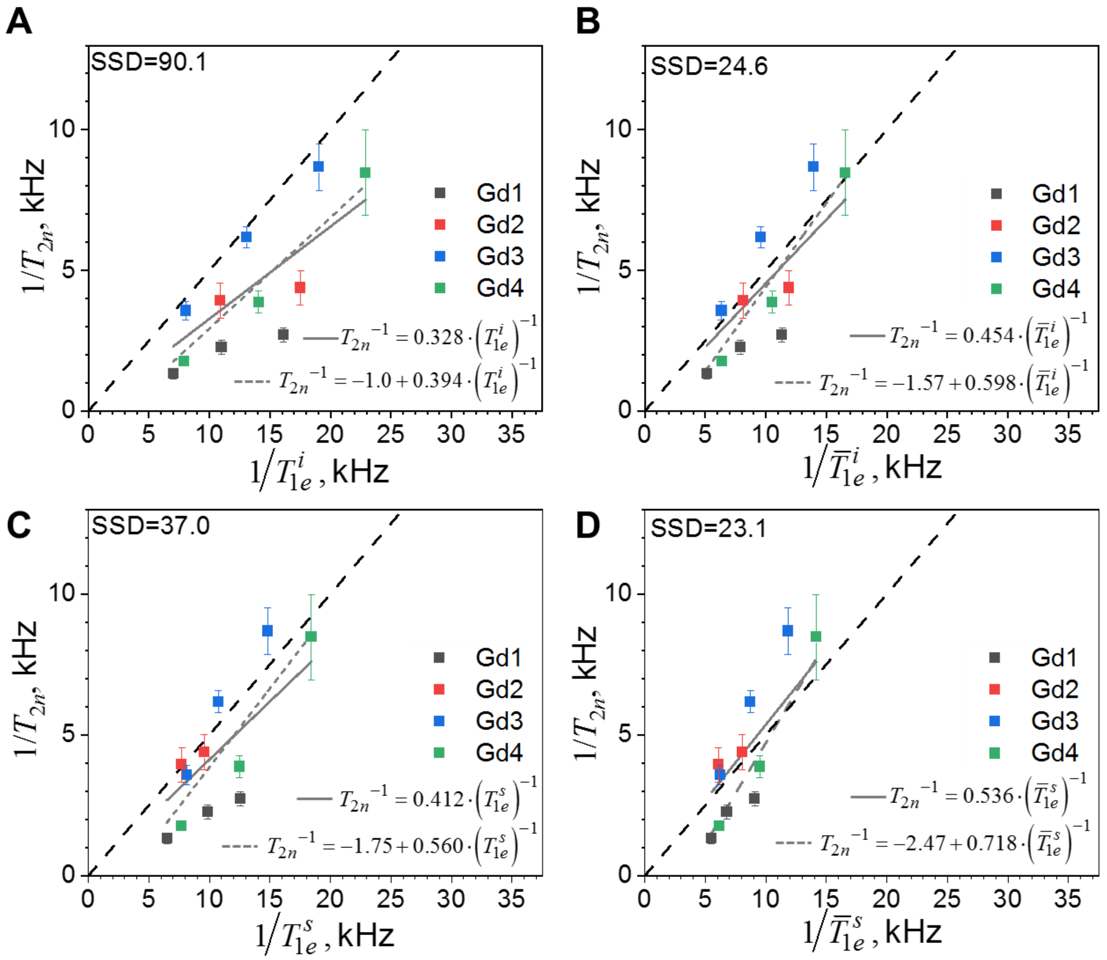

Figure S8. ^{19}F $1/T_{2n}$ as a function of $1/T_{1e}$ measured using inversion recovery (**A, C**) and saturation recovery (**B, D**) experiments. The values in panels (**C, D**) correspond to mean values determined by the first moments of the stretched exponentials. The sum of squared deviation (SSD, in kHz^2) of the experimental points from the equation $T_{2n}{}^{-1} = (2T_{1e})^{-1}$ (shown as a dashed line) is displayed in each panel. The linear regression of the data is shown as gray solid lines (intercept set to be zero) and gray short-dashed lines (free intercept), and regression equations with optimal parameters are listed on each panel. In the latter case, negative intercept values are obtained, which are not physically meaningful. Hence, the models with zero intercept were considered more reliable.

S7. ^{1}H nuclear spin echo decay in Gd1 and Gd3

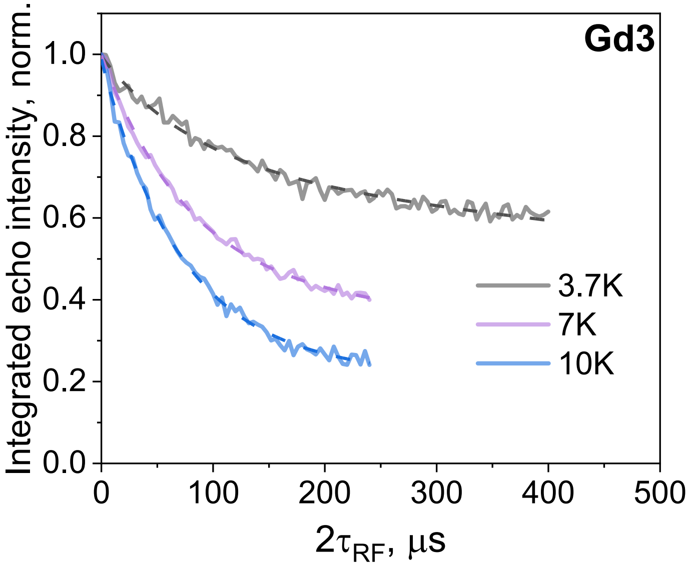

Figure S9. NSE decays of ^{1}H in **Gd3** recorded at $\nu_{RF}=\nu_I-0.47$ MHz, at temperatures in the range 3.7–10 K, solid lines – experimental data, dashed lines – biexponential fits with parameters listed in **Table S4**.

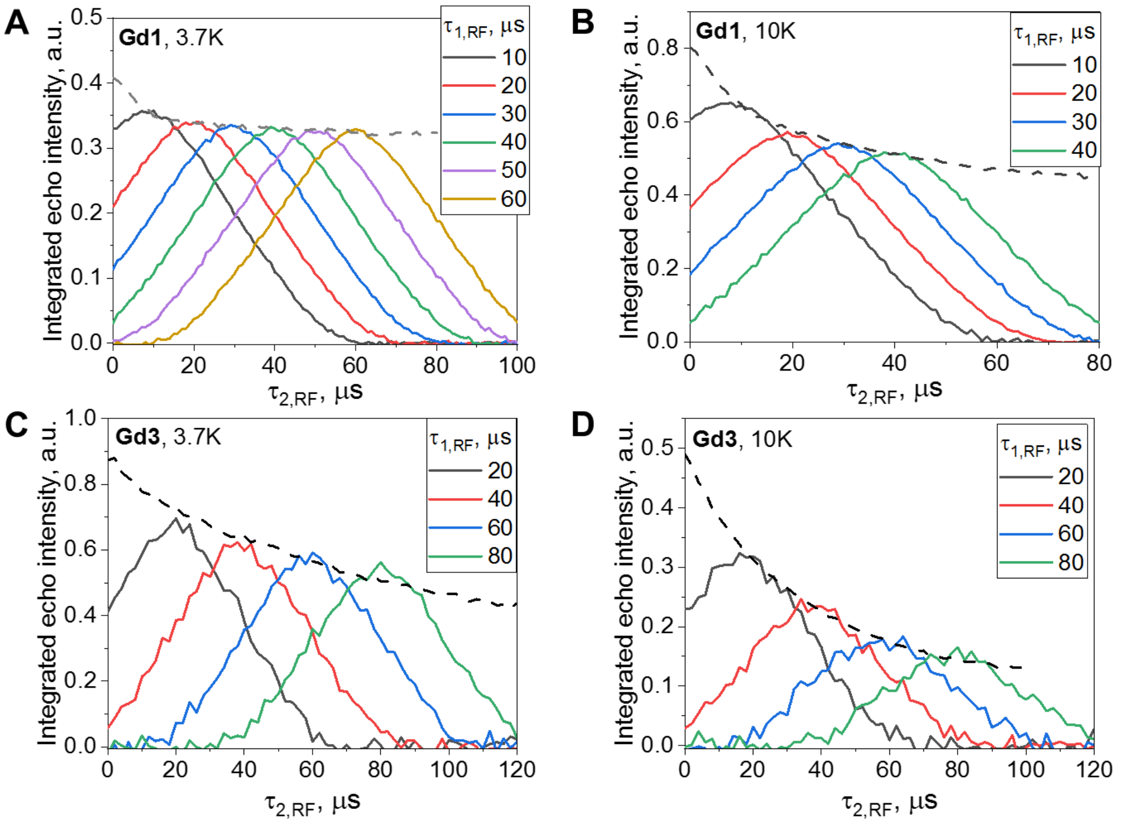

Figure S10. NSEs (solid lines) and NSE decay curves (dashed lines) for ^{1}H nuclei in **Gd1** (**A, B**) and **Gd3** (**C, D**) recorded at ν_{RF}=ν_I–0.47 MHz at 3.7 K (**A,C**) and 10 K (**B, D**).

Table S4. Results of biexponential fitting parameters of ^{1}H NSE decay traces for **Gd1** and **Gd3** at 3.7–10 K.

	T, K	$\bar{T}_{1e}^{s}$, μs	$1/\bar{T}_{1e}^{s}$, kHz	T_{2n} fast, μs	$1/T_{2n}$ fast, kHz	T_{2n} slow, μs	$1/T_{2n}$ slow, kHz	Fraction of fast
**Gd1**	3.7	180	5.54	13.7±0.8	73.1	2900±250	0.34	0.18±0.01
**Gd1**	7	148	6.78	20.8±0.8	48.0	715±30	1.4	0.26±0.01
**Gd1**	10	110	9.11	20.8±0.8	48.0	595±20	1.7	0.29±0.01
**Gd3**	3.7	161	6.21	85.0±0.7	11.8	2000^a	0.5	0.27±0.02
**Gd3**	7	114	8.74	78±2	12.8	2000^a	0.5	0.57±0.01
**Gd3**	10	84.3	11.9	70±2	14.4	2000^a	0.5	0.74±0.02

^{a} the value was not varied

S8. ¹H ENDOR spectra and NSE decays of Gd1 in deuterated and partially protonated solvent

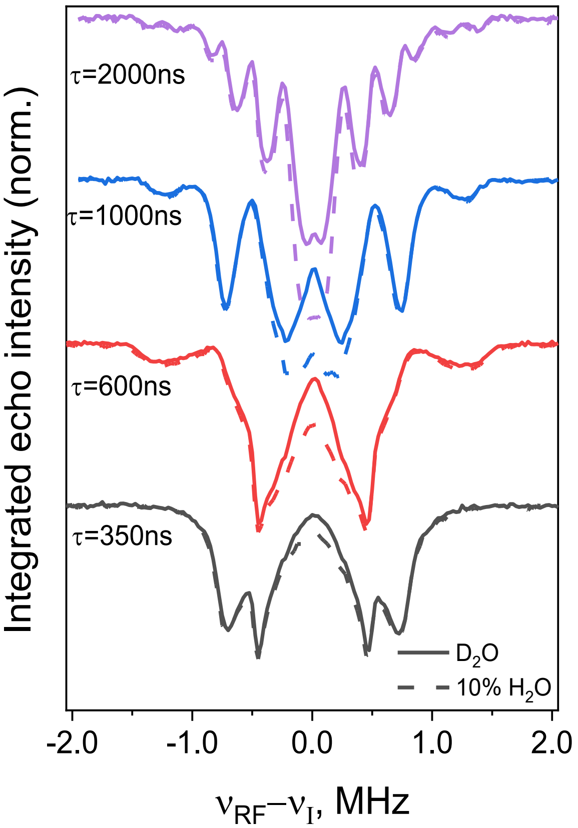

Figure S11. ^{1}H ENDOR spectra of **Gd1** at 10 K in fully deuterated solvent (solid lines) and in the presence of 10 vol. % H_2O (dashed lines).

Table S5. Parameters of biexponential fitting of ^{1}H NSE decay traces of **Gd1** at 10 K recorded for different ENDOR lines and different degrees of matrix protonation. Relaxation times of fast and slow components were fixed to be the same as listed in **Table S4**, and relative fractions of the fast relaxation component were varied.

vol. % H_2O	*a*, MHz	T_{2n} fast, μs	T_{2n} slow, μs	Fraction of fast
0	1.44	20.8	595	0.29±0.01
	0.94			0.31±0.01
	~0			0.46±0.02
10	1.44	20.8	595	0.28±0.02
	0.94			0.31±0.01
	~0			0.67±0.03

Supplementary references

(S1) Yang, Y.; Yang, F.; Gong, Y.-J.; Bahrenberg, T.; Feintuch, A.; Su, X.-C.; Goldfarb, D. High Sensitivity in-Cell EPR Distance Measurements on Proteins Using an Optimized Gd(III) Spin Label. *J. Phys. Chem. Lett.* **2018**, *9* (20), 6119-6123. DOI: https://doi.org/10.1021/acs.jpclett.8b02663.

(S2) Bogdanov, A.; Frydman, V.; Seal, M.; Rapatskiy, L.; Schnegg, A.; Zhu, W.; Iron, M.; Gronenborn, A. M.; Goldfarb, D. Extending the Range of Distances Accessible by 19F Electron–Nuclear Double Resonance in Proteins Using High-Spin Gd(III) Labels. *J. Am. Chem. Soc.* **2024**, *146* (9), 6157-6167. DOI: https://doi.org/10.1021/jacs.3c13745.

(S3) Laurent, S.; Vander Elst, L.; Galaup, C.; Leygue, N.; Boutry, S.; Picard, C.; Muller, R. N. Bifunctional Gd(III) and Tb(III) Chelates Based on a Pyridine–Bis(Iminodiacetate) Platform, Suitable Optical Probes and Contrast Agents for Magnetic Resonance Imaging. *Contrast Media & Molecular Imaging* **2014**, *9* (4), 300-312. DOI: https://doi.org/10.1002/cmmi.1576.

(S4) Feintuch, A.; Shimon, D.; Hovav, Y.; Banerjee, D.; Kaminker, I.; Lipkin, Y.; Zibzener, K.; Epel, B.; Vega, S.; Goldfarb, D. A Dynamic Nuclear Polarization Spectrometer at 95 GHz / 144 MHz with EPR and NMR Excitation and Detection Capabilities. *J. Magn. Reson.* **2011**, *209* (2), 136-141. DOI: https://doi.org/10.1016/j.jmr.2010.12.010.

(S5) Mentink-Vigier, F.; Collauto, A.; Feintuch, A.; Kaminker, I.; Tarle, V.; Goldfarb, D. Increasing Sensitivity of Pulse EPR Experiments Using Echo Train Detection Schemes. *J. Magn. Reson.* **2013**, *236*, 117-125. DOI: https://doi.org/10.1016/j.jmr.2013.08.012.

(S6) Epel, B.; Arieli, D.; Baute, D.; Goldfarb, D. Improving W-Band Pulsed ENDOR Sensitivity—Random Acquisition and Pulsed Special Triple. *J. Magn. Reson.* **2003**, *164* (1), 78-83. DOI: https://doi.org/10.1016/S1090-7807(03)00191-5.

(S7) Dennis, J. E.; Gay, D. M.; Walsh, R. E. An Adaptive Nonlinear Least-Squares Algorithm. *ACM Trans. Math. Softw.* **1981**, *7* (3), 348–368. DOI: 10.1145/355958.355965.

(S8) Nehring, J.; Saupe, A. Anisotropies of the 19F Chemical Shifts in Fluorobenzene Compounds from NMR in Liquid Crystals. *J. Chem. Phys.* **1970**, *52* (3), 1307-1310. DOI: https://doi.org/10.1063/1.1673130.

(S9) Grage, S. L.; Dürr, U. H. N.; Afonin, S.; Mikhailiuk, P. K.; Komarov, I. V.; Ulrich, A. S. Solid State 19F NMR Parameters of Fluorine-Labeled Amino Acids. Part Ii: Aliphatic Substituents. *J. Magn. Reson.* **2008**, *191* (1), 16-23. DOI: https://doi.org/10.1016/j.jmr.2007.11.016.
